# Whole-Genome Deep Sequencing of the Healthy Adult Nasal Microbiome

**DOI:** 10.3390/microorganisms12071407

**Published:** 2024-07-12

**Authors:** Mark Cannon, Gustavo Ferrer, Mari Tesch, Matthew Schipma

**Affiliations:** 1 Ann & Robert H. Lurie Children’s Hospital of Chicago, Chicago, IL 60611, USA; 2 Feinberg School of Medicine, Northwestern University, Chicago, IL 60611, USA; 3 Aventura Hospital Pulmonary and Critical Care Fellowship, Aventura, FL 33180, USA; gferrer@pulmonary-institute.com (G.F.); research@drferrerbiopharma.com (M.T.); 4 QDSC, NUSeq Core, Northwestern University, Chicago, IL 60611, USA; m-schipma@northwestern.edu

**Keywords:** deep sequencing, nasal microbiome, gateway microbiome, diversity, richness, probiotics, nasal spray

## Abstract

This study aimed to determine shifts in microbial populations regarding richness and diversity from the daily use of a popular over-the-counter nasal spray. In addition, the finding of nasal commensal bacterial species that overlap with the oral microbiome may prove to be potential probiotics for the “gateway microbiomes”. Nasal swab samples were obtained before and after using the most popular over-the-counter (OTC) nasal spray in 10 participants aged 18–48. All participants were healthy volunteers with no significant medical histories. The participants were randomly assigned a number by randomizing software and consisted of five men and five women. The sampling consisted of placing a nasal swab atraumatically into the nasal cavity. The samples were preserved and sent to Northwestern University Sequencing Center for whole-genome deep sequencing. After 21 days of OTC nasal spray use twice daily, the participants returned for further nasal microbiome sampling. The microbial analysis included all bacteria, archaea, viruses, molds, and yeasts via deep sequencing for species analysis. The Northwestern University Sequencing Center utilized artificial intelligence analysis to determine shifts in species and strains following nasal spray use that resulted in changes in diversity and richness.

## 1. Introduction

The number of patients with an allergic response to their environment is increasing and is now more than 40% in numerous populations in the United States and Europe [1]. Allergic rhinitis refers to an allergic response that occurs in the nose [2]. The number of patients suffering from allergic rhinitis, commonly known as hay fever, in the United States is approximately 30% [3]. Allergic rhinitis contributes to lost or unproductive time at work and school, sleep problems, and reduced participation in outdoor activities. The ability to control asthma and allergic rhinitis in individuals with asthma has been linked to the control of allergic rhinitis [4].

According to published studies, most individuals with asthma develop rhinitis [5]. In addition, the presence of allergic rhinitis (seasonal or perennial) significantly increases the likelihood of asthma; up to 40% of people with allergic rhinitis have or will have asthma [6]. It is also essential to define how breathing is affected in patients with obstructive sleep apnea (OSA) [7]. Breathing starts at the nose, and while OSA is characterized by the collapse of the muscles of the oropharyngeal airways, nasal obstruction and OSA are usually co-existing conditions that worsen each individual condition [8,9,10]. Therapies to improve airflow through the nose in compromised patients significantly reduce daytime and nighttime symptoms [11]. The nose is responsible for almost 50% of the resistance when transporting air from the nose to the lungs and plays an essential role in humidification, heating, and air filtration [12]. The tissues inside the nose, known as the nasal mucosa, are dynamic organs regulated by the autonomic nervous system [13]. Periodic nasal congestion and decongestion are termed the “nasal cycle” [14]. In patients with a permanent one-sided nasal obstruction, the nasal cycle can make breathing air into the body difficult [15].

Nasal sprays are used by a large percentage of the population, especially with recent news reports demonstrating the inhibitory action of xylitol nasal sprays and other ingredients on the attachment (then endocytosis) of SARS-CoV-2 virus to nasal cells [16,17,18]. In addition, xylitol has also been reported to inhibit the oncogenesis of oral cells, making xylitol nasal sprays even more desirable for this population [19]. However, no study has analyzed the potential shift in the nasal microbiome after using xylitol nasal spray.

Nasal and lung microbiomes may be affected by the gut microbiome [20]. Formula-fed infants are at an increased risk of infections [21]. Owing to the crosstalk between the mucosal systems of the gastrointestinal and respiratory tracts, adding synbiotics (prebiotics and probiotics) to infant formulas may prevent infections even at distant sites [22,23]. In a study on infants who were born full-term and weaned from breast milk, they were randomized to either prebiotic formula (fructo- and galactooligosaccharides) or the same prebiotic formula along with *Lactobacillus paracasei* ssp. *paracasei* F19 (synbiotics) from 1 to 6 months of age. Synbiotic feeding led to reduced Klebsiella, enrichment of bifidobacteria, and increases in microbial degradation metabolites implicated in immune signaling and in the gut–lung and gut–skin axes [23]. With the synbiotic effects on gut microbiota development, *L. fermentum* PCC and *L. reuteri* RC-14 were more resistant to gastric conditions, and their survival rate was further improved in the presence of 5 out of 10 tested pectins. Additionally, two pectins positively affected the viability of the less resistant *L. rhamnosus LGG* and *L. paracasei* F-19 [24].

Since the advent of high-throughput sequencing, PCR-amplified 16S sequences have typically been clustered based on their similarity to generate operational taxonomic units (OTUs), and representative OTU sequences have been compared with reference databases to infer the likely taxonomy. However, convenient and powerful usage of 16S rRNA has necessitated certain assumptions, such as the now historical assumption that sequences of >95% identity represent the same genus. In contrast, sequences with >97% identity represent the same species [25,26].

Objective:

The principal objective of this study was to discover the normal complete nasal microbiome after using an over-the-counter nasal spray, which, as of this date, has yet to be completed. In addition, more information is needed on the microbiome shifts that occur in response to xylitol nasal spray. Before the widespread use of nasal sprays, what may be considered normal should be determined. Shifts in microbial diversity and richness should be regarded as likely.

## 2. Materials and Methods

This study performed a nasal swab test before and after using the most popular OTC nasal spray in 10 participants aged 18–48. All participants had non-significant medical histories with no history of allergies or medication use that could affect the nasal microbiome. The participants consisted of five men and five women who were randomly assigned a number using randomizing software. The participants signed an informed consent form (submitted to the IRB and accepted), and the principal investigator kept all personal protected information in a hard copy safely locked in a secure cabinet.

An e-mail newsletter recruited participants from all patients of the principal investigator, and recruiting posters were placed in the reception area of the PI office. No inducements or compensation were provided to the participants. The study protocol, informed consent, medical history, and sampling are discussed separately. The samples were preserved and shipped by FedEx to Northwestern University’s NUSeq Center for Whole Genome Deep Sequencing. After 21 days of OTC nasal spray use twice daily, the participants returned for another sampling of their nasal microbiome, which FedEx then shipped to NUSeq for deep sequencing. The participants were instructed to use the spray before dismissal during the first visit. The spray was obtained as samples normally distributed to clinics at no charge and was already in the principal investigator’s inventory. These samples were often administered to pediatric patients diagnosed with sleep apnea.

Sampling consisted of placing a nasal swab atraumatically into the anterior nasal cavity, similar to self-performed sampling for COVID-19 tests. No injury was reported, and only slight discomfort, if any, was reported. The swab was placed approximately one inch into the nares, rotated for 15 s, and then placed into the other nares for another rotation of 15 s. The swabs were placed in a preservative and sealed safely. Biosafety envelopes were used for shipping according to previous protocols. After the second sampling, patients were excluded from the study. All personal information was destroyed after two years.

Xlear Sinus Care is an OTC xylitol solution. Xylitol is a natural sugar (pentose aldose) that inhibits biofilm formation [27]. Pure xylitol is a white crystalline substance in many fruits and vegetables [28]. According to the manufacturer, the Xlear solution cleanses, hydrates, dries, and irritates tissues. Xlear products use a patented xylitol solution that inhibits bacteria and pulls moisture into the nasal cavity. According to recently published research, xylitol also inhibits the viral invasion of cells [17].

Ingredients:

- Purified water;
- Xylitol;
- Saline;
- Grapefruit seed extract.

Study Data:

Microbiome Analysis. The quality of the reads in the FASTQ format was evaluated using FastQC (v1.12.1). Reads were trimmed to remove Illumina adapters from the 3’ ends using Cutadapt (3.7) [29]. Paired-end trimmed reads were aligned using Kraken2 (2.1.2) with default parameters, except for using 12 threads instead of the default value of 1 [30]. The standard Kraken2 database includes archaea, bacterial, viral, plasmid, and human genomes. Taxa were determined using the standard database, which was loaded using kraken2-build. Alignments that could not be classified into known taxa were excluded. Taxon abundance estimates were performed using Bracken (2.7) with default parameters, except for using 12 threads instead of the default value of 1 [31]. Normalization and differential expression were calculated using DESeq2 (1.14.1) [32]. The libraries were constructed using Nextera kits according to the manufacturer’s instructions (Illumina, San Diego, CA, USA). The samples were sequenced paired-end 150 base pairs on the NovaSeq 6000 instrument (Illumina, San Diego, CA, USA). The total sequencing depth was nearly 2.3 billion reads with an average of 47 million reads per sample. Default parameters were used to improve standardization and remain within NUseq Center protocols. Data reside at the NUSeq Center at Northwestern University, and the nasal samples are preserved for future additional analysis.

## 3. Results

The analysis determined 2558 taxa with greater abundances of the following top taxa: *Xanthobacter autotrophicus*, *Streptomyces* sp. *ICC1*, *Streptomyces armeniacus*, *Streptococcus thermophilus*, *Streptococcus sanguinis*, *Streptococcus pyogenes*, *Streptococcus oralis*, *Staphylococcus epidermidis*, *Staphylococcus aureus*, *Salmonella enterica*, *Rothia aeria*, *Ralstonia pickettii*, *Pseudomonas aeruginosa*, *Peptoniphilus harei*, *Lawsonella clevelandensis*, *Klebsiella pneumoniae*, *Finegoldia magna*, *Escherichia coli*, *Enterococcus faecium*, *Dolosigranulum pigrum*, *Delftia lacustris*, *Cutibacterium granulosum*, *Cutibacterium acnes*, *Corynebacterium tuberculostearicum*, *Corynebacterium striatum*, *Corynebacterium segmentosum*, *Corynebacterium propinquum*, *Corynebacterium macginleyi*, *Corynebacterium kefirresidentii*, *Corynebacterium glutamicum*, *Corynebacterium diphtheriae*, *Burkholderia dolosa*, *Brevibacillus brevis*, *Bartonella krasnovii*, *Anaerococcus prevotii*, *Aeromonas caviae*, and *[Haemophilus] ducreyi*.

The routine use of xylitol nasal spray changed the relative abundance of ten taxa (Table 1). This result is not surprising because recently published research has demonstrated that even a few species being changed have a cascading effect throughout the microbiome [23]. Xylitol should reduce the number of pathogenic bacteria, which would also affect the pathogens’ synergistic co-pathogens [33,34,35]. Reports have previously been published that the oral microbiome has a “downstream effect” on the gut microbiome [36,37]. It would not be surprising if the nasal gateway microbiome did not have the same effect, with a reduction in pathogens of the anterior nasal passageway strain/species shifting the respiratory microbiome.
*Akkermansia muciniphila**Rhodococcus qingshengii**Streptomyces* sp. *ICC1**Streptomyces armeniacus**Actinomyces* sp. *oral taxon 414**Klebsiella oxytoca**Acinetobacter ursingii**Klebsiella variicola**Enterobacter hormaechei**Brevundimonas diminuta*

**Figure 1:**
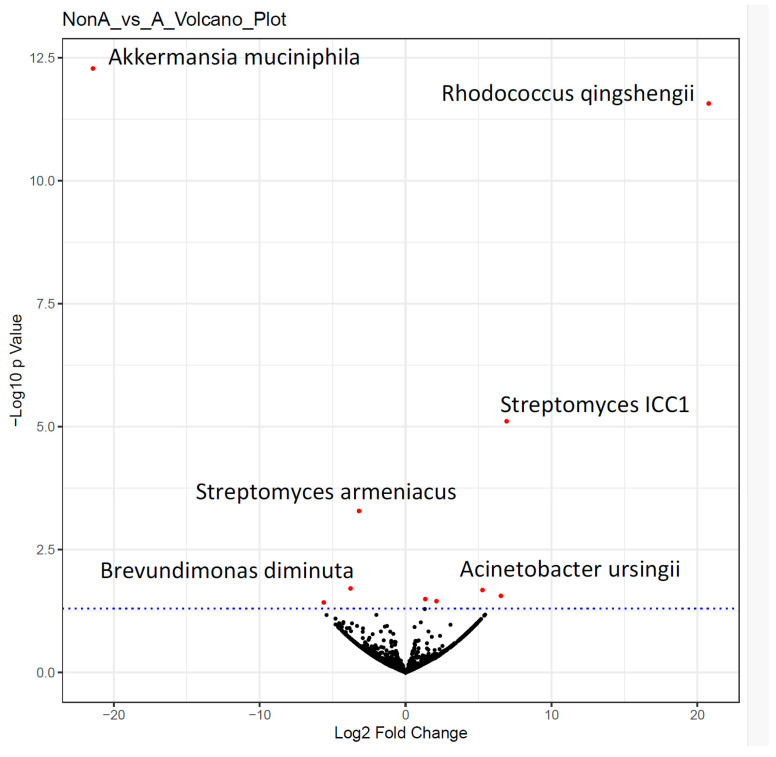
Non-A_vs._A Volcano Plot demonstrating microbiome shift from control to xylitol-treated nasal microbiome. This is a labeled volcano plot that plots the *p*-values against the log fold change. Red dots are considered significantly different. The further a dot is away from the origin, the more significant the difference. Any taxa above the blue dotted line displayed significant changes after nasal spray use.

Four species decreased, with six increasing abundance (Figure 1. Volcano plot of A versus non-A, after xylitol and before xylitol). *Rhodococcus qingshengii* (soil organism with anti-fungal properties) significantly increased, but *Akkermansia muciniphilia* substantially decreased. *Akkermansia muciniphilia* is considered a probiotic in the gut and the oral cavity. *Brevundimonas diminuta* was also decreased and is ubiquitous in humans but has been associated with infections in immunocompromised and cancer patients. *Acinetobacter ursingii* increased in predominance and has been associated with immunocompromised patient infections (see Table 1). It is essential to note that almost all microbes may cause an illness if in the wrong place at the wrong time. We lack sufficient knowledge of the nasal gateway microbiome to properly judge which bacteria are acceptable commensals and which are pathobionts. For instance, *Streptomyces armeniacus* is a spore-forming soil organism, a probiotic producing Streptopyrrole, and the abundance is increased by xylitol therapy. The presence of so many soil-based microorganisms is to be expected as they may become airborne via dust particles. One can only imagine the bacterial load a runner takes in nasally as they sprint on a dirt path. The Streptopyrrole probiotic bacteria *Streptomyces armeniacus* may be an essential link in nasal health.

The greater majority of taxa were bacterial, although these viruses were more predominant, Pahexavirus ATCC29399BT, Propionibacterium phage BruceLethal (infects *Propionibacterium acnes*), Streptococcus phage IPP9, and Streptococcus phage phiARI0455b. The fungus *Puccinia graminis*, which causes significant disease in cereal crops, was also found in the samplings. Crop species affected by the disease include bread wheat, durum wheat, barley, and triticale. Another fungus was *Candida tropicalis*, a pathogen similar to *Candida albicans* and capable of forming a pathogenic biofilm. *Blastocytis hominis*, a single-cell parasite responsible for gastrointestinal infections, but with 22 strains, of which only nine can cause infections, was also isolated. *Methanosarcina mazei* an anaerobic archaeon that can metabolize various substrates and produce methane was present. The 50 most abundant taxa are listed in Table 2.

**Figure 2:**
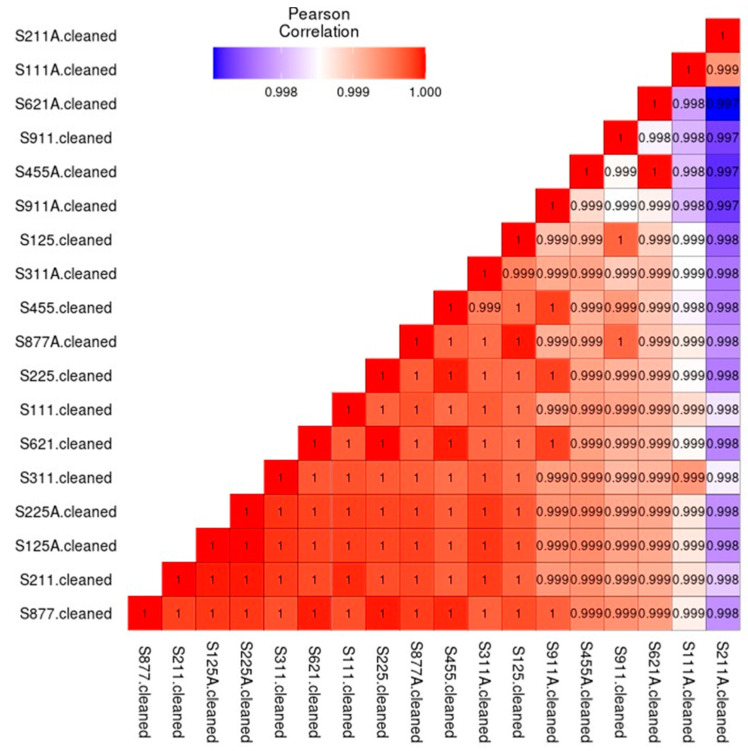
Pearson Correlation demonstrates the statistical differences between the A and non-A groups. In this figure, A refers to subjects after using a xylitol spray for 21 days. This is a correlation matrix displaying the R-squared value of the correlation between each pair of samples. A perfect score is 1.0; the higher the score, the more similar the samples. Negative scores mean negative correlations; that is, the microbe abundances are high in one sample and low in the other. Cleaned refers to the samples having all contaminants and human DNA removed.

**Figure 3:**
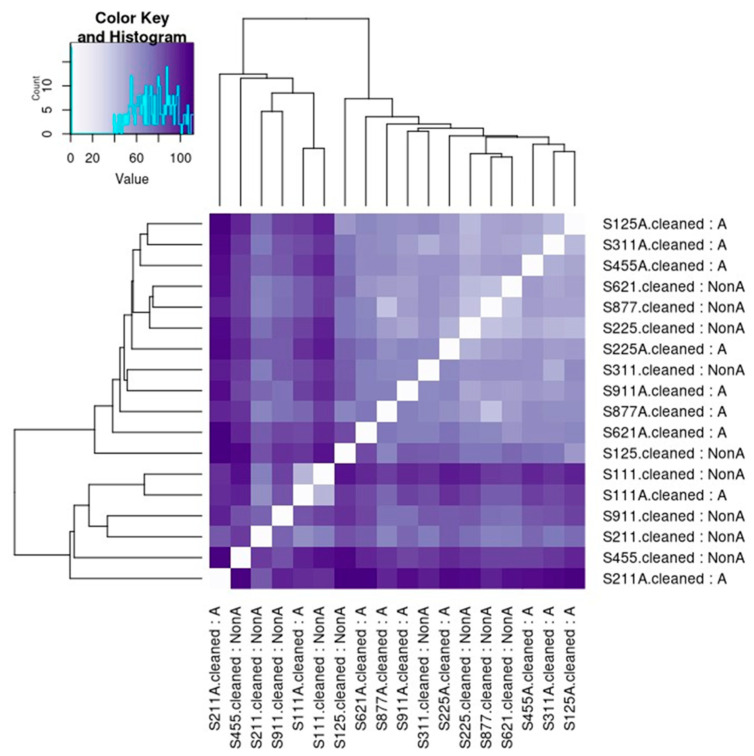
Heatmap demonstrating the differences between the A and non-A groups. Non-A is before the use of the xylitol spray. Cleaned means that all containment has been removed, especially human DNA. This is a heatmap showing the similarity between samples, similar to a correlation matrix. The Euclidean distance is computed between each pair of samples, and the distribution of distances is shown in the legend of the upper left. The lighter the shade of purple, the smaller the distance. The smaller the distance, the more similar the samples are. This graph shows an extra feature of clustering the samples based on similarity. Even though both are shown, the x and y axes are redundant.

Pearson correlation demonstrated statistical differences between the A and Non-A groups. A group refers to the subjects using a xylitol spray for 21 days, non-A was the group before using the spray. A and non-A nomenclature was used to blind the laboratory personnel to the nature of the research (see Figure 2). Similar to a correlation matrix, a heatmap demonstrated the differences between the A and non-A groups (see Figure 3). 

## 4. Discussion

The microbial analysis included all bacteria, archaea, viruses, molds, and yeasts obtained via deep sequencing for species analysis. Artificial intelligence analyses by the Northwestern University Sequencing Center revealed shifts in species and strains due to the use of the OTC nasal spray, resulting in changes in diversity or richness. Deep sequencing and artificial intelligence analysis may be used to determine changes in Richness and Shannon diversity. Genus, species, and strain shifts determined the precise response of the nasal microbiome to the most common OTC nasal spray. Moreover, the complete nasal microbiome by deep sequencing was yet to be discovered. In addition, more information was needed on the microbiome shifts that occur in response to xylitol nasal spray. Before the widespread use of COVID-19 precautions, what may be considered the normal nasal microbiome needed to be determined. The nasal microbiome of healthy adults before and after xylitol exposure has yet to be analyzed using whole-genome deep sequencing methods.

The large number of taxa found may not represent the total number likely present in the nasal cavity. The study ended at the number reported mainly owing to its economic restraints and the use of artificial intelligence with machine language development, which demanded technology resources. In addition, there are often constraints owing to the availability of DNA libraries for comparison. Regardless of these constraints, we found that routine xylitol nasal spray use significantly affects the prevalence of at least ten taxa.

Several probiotic bacteria have been identified in the nasal cavity; oral or gut probiotics are often abundant. The nasal microbiome also contains several skin commensals, such as *Staphylococcus epidermidis* and *Cutibacterium acnes*. This indicates that the nasal microbiome combines the features of both the oral and skin microbiomes. Additionally, bacteria are often specific to the nasal cavity. Previous studies have shown that the most abundant bacteria are *Staphylococcus aureus*, *Staphylococcus epidermidis*, and *Propionibacterium acnes* [38,39].

Of evolutionary importance, *Bartonella kraznovii* is associated with sub-Saharan black African rats [40]. During the great “throttling”, *Homo sapiens* moved into caves along the seacoast because of significant climate changes (Marine Isotope Stage 6) [41]. The surviving H. sapiens’ diet included tubers, often roasted in caves [42]. The roasted tubers were consumed as a survivor’s food, but any leftovers would have been available for the cave rodents. The association of this nasal bacterium with rodents that share *Homo sapiens* caves should not be surprising and is an interesting result of this study.

*Burkholderia dolosa* is a species of bacteria member of the *Burkholderia cepacia* complex. This strain is highly drug-resistant and primarily found in immunocompromised patients [43]. *B. dolosa* chronic infection in cystic fibrosis is associated with an accelerated loss of lung function and decreased survival [44]. However, this was observed in several participants. Another rare pathogen found in patients with cystic fibrosis, *Achromobacter xylosoxidans*, was also found in a few participants [45]. However, as in all diseases, it is not just the presence of pathogens but the absence of compensatory commensals and probiotic microorganisms that influence the expression of pathology [46]. Xylitol solutions may inhibit pathogens without negatively reducing the levels of probiotic bacteria, as has often been reported in the literature [47]. Xylitol nebulizers have proven effective in patients with cystic fibrosis [48].

Nasal microbiome changes and sleep-disturbed breathing may be correlated, which warrants significant research [49]. This research project utilized young adults, not children, and examined the possible correlation between the nasal microbiome and nasal obstruction due to chronic inflammation, which leads to nasal airway obstruction [50]. The connection between the airway and microbiome has been well established. Although many researchers believe that the airway affects the microbiome, they do not consider that it affects respiration, albeit indirectly. For instance, the oral microbiome produces nitrites from nitrates, eventually processed by stomach acid into nitric oxide [51]. Salivary nitric oxide inhibits decay, reduces periodontal disease, lowers CRP, and potentially affects airway resistance [52]. Systemic serum levels are associated with normosystolic blood pressure during pregnancy [53]. Nitrate-reducing oral bacterial levels are also linked to a normal pregnancy, and oral dysbiosis due to *Porphyromonas gingivalis* causes pre-eclampsia [54,55,56].

**Figure 4:**
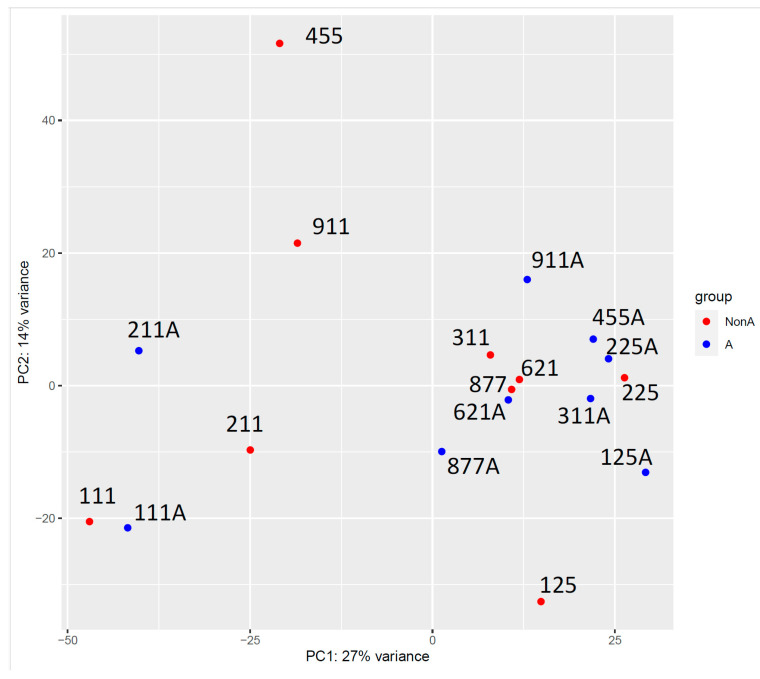
This is a PCA (principal components analysis) plot that shows the similarities between samples, the nearer samples are on the plot, the more similar. Each sample is labeled.

Ideally, future studies should use larger population samples to examine the association between diseases and microbiome shifts. The first step in our study was to analyze the healthy microbiome before and after a 21-day course of xylitol (see Figure 4). All patients were asymptomatic; therefore, no clinical correlations could be established. Animal studies have demonstrated an association between the gut and feline upper respiratory tract disease, specifically in felines. FURTD, often caused by infectious etiologies, is a multifactorial syndrome that affects feline populations. The eco-phylogenetic method identified 136 and 89 microbial features, respectively, within the gut and nasal microbiomes, significantly associated with active FURTD clinical signs [57]. Nasal and gut microbial community members are associated with a chronic clinical course [58]. Studies have shown that endogenous microbiome dysbiosis can affect mucosal health and disease severity. Some bacterial species exhibit protective properties, whereas others are pathogenic [59]. Antimicrobial agents can create a similar disruption, affect the nasal microbiome balance, and provoke allergic responses [60]. Probiotics offer a promising avenue for developing systemic and topical therapies to strategically manipulate the biological host load, thereby augmenting immune homeostasis [61]. Combining probiotics indigenous to oral and nasal cavities with prebiotics, such as xylitol, may inhibit pathogens and restore nasal health. This combination therapy may benefit the developing fetus and infant from maternal usage and prevent pathogenic microbiome development [62,63,64]. An example of a potential nasal probiotic therapy is the discovery that *Lactobacilli* taxa can be present on the facial skin, in the nasal cavity, and in the vagina. Hypothetically, multiple microniches provide commensal protection to multiple microniches [65]. Further discovery that *Lactobacilli casei* (AMBR2) is present in healthy individuals but not in those with chronic rhinosinusitis led to the new designation of “keystone” probiotic commensal [66].

Because so many other respiratory illnesses are associated with a dysbiotic nasal microbiome, discovering a eubiotic nasal microbiome should be an important objective [3,4,5,6]. A study by Chin et al. showed that the upper airway microbiome is associated with airway inflammation disorders and the level of asthma control [67]. This would indicate that the goal of healthcare professionals should always be not just to treat symptoms but to re-establish an eubiotic nasal microbiome [68]. This treatment could be started in infancy and then successfully prevent later respiratory disease [69]. The knowledge of what constitutes a healthy nasal microbiome in symptomless young adults and how to maintain the microbiome is, therefore, very significant. The present study helps provide the necessary data on what is bacterially present in asymptomatic young adults.

Another important aspect of the nasal microbiome is its importance in overall health. Nasal vaccines may prevent many respiratory diseases by increasing the IgA antibody response and improving mucosal defenses [70]. Nasal vaccines induce protective immunity that prevents the invasion of pathogens from mucosal surfaces and inhibits severe disease. Nasal vaccines are most appropriate for respiratory infectious diseases because they induce effective immunity in the upper and lower respiratory tracts [71]. Intranasal vaccination induces the nasal IgA antibody that protects against respiratory viruses like influenza and SARS-CoV-2 [72]. Using commensal bacteria with hemagglutinin in nasal vaccines increased the antibody response [73,74]. Future nasal vaccines may utilize newly discovered nasal commensal bacteria as adjutant carriers of the antigen that stimulates the protective immune response.

## 5. Conclusions

The nasal microbiome is rich and diverse, containing taxa often associated with oral and skin microbiomes. Microbial interventions, even with OTC nasal rinses, may significantly affect the nasal microbiome, and associated microbiomes.

## Figures and Tables

**Table 1:** The relative abundance of these ten taxa was significantly different after using the xylitol nasal spray. Four species decreased, and six increased in abundance. *Rhodococcus qingshengii* (soil organism with anti-fungal properties) significantly increased, but *Akkermansia muciniphilia* substantially decreased. *Brevundimonas diminuta* was also decreased.

Microbe	log2FoldChange	lfcSE	Stat	*p* Value
*Akkermansia muciniphila*	−21.42441822	2.967913092	−7.218681128	5.25 × 10^−13^
*Rhodococcus qingshengii*	20.77410089	2.97113001	6.991986491	2.71 × 10^−12^
*Streptomyces* sp. *ICC1*	6.922848082	1.548527233	4.470601442	7.80 × 10^−6^
*Streptomyces armeniacus*	−3.191007111	0.919334063	−3.470998453	0.000518527
*Actinomyces* sp. *oral*	−3.771603944	1.615989861	−2.333927976	0.019599494
*Klebsiella oxytoca*	5.272476299	2.286510228	2.305905408	0.021115918
*Acinetobacter ursingii*	6.534219931	2.968012555	2.201547268	0.027697304
*Klebsiella variicola*	1.349165754	0.630129643	2.141092342	0.032266592
*Enterobacter hormaechei*	2.117319005	1.007575689	2.101399458	0.035605916
*Brevundimonas diminuta*	−5.607547967	2.699838683	−2.076993712	0.03780214

**Table 2:** The list is of the top 50 most common taxa, with bacterial species being predominant. Two key oral probiotics, *Streptococcus oralis* and *Streptococcus sanguinis*, are present in the nasal microbiome.

*Staphylococcus aureus*
*Vibrio* sp. *Scap24*
*Corynebacterium segmentosum*
*Aeromona scaviae*
*Cutibacterium acnes*
*Salmonella enterica*
*Streptomyces* sp. *ICC1*
*Dolosigranulum pigrum*
*Klebsiella pneumoniae*
*Delftia lacustris*
*Corynebacteriumm kefirresidentii*
*Corynebacterium propinquum*
*Staphylococcus epidermidis*
*Finegoldia magna*
*Corynebacterium tuberculostearicum*
*Lawsonella clevelandensis*
*Corynebacterium macginleyi*
*Bartonella krasnovii*
*Peptoniphilus harei*
*Cutibacterium granulosum*
*Pseudomonas aeruginosa*
*Enterococcus faecium*
*Xanthobacter autotrophicus*
*Aeromonas hydrophila*
*Haemophilus ducreyi*
*Streptococcus mitis*
*Burkholderia dolosa*
*Xanthomonas citri*
*Staphylococcus capitis*
*Klebsiella grimontii*
*Escherichia coli*
*Cutibacterium avidum*
*Corynebacterium striatum*
*Klebsiella variicola*
*Streptomyces armeniacus*
*Enterococcus faecalis*
*Delftia acidovorans*
*Citrobacter koseri*
*Alistipes communis*
*Streptococcus oralis*
*Corynebacterium diphtheriae*
*Actinomyces oris*
*Streptococcus pneumoniae*
*Mycolicibacter* sp. *MYC340*
*Streptococcus sanguinis*
*Micrococcus luteus*
*Ralstonia pickettii*
*Mycolicibacterium austroafricanum*
*Bacillus* sp. *BD59S*

## Data Availability

Due to the need to maintain strict privacy of the subjects (children), data were protected, and consent contained specific language assuring complete safeguarding measures.
